# Effects of Low-Dose and Long-Term Treatment with Erythromycin on Interleukin-17 and Interleukin-23 in Peripheral Blood and Induced Sputum in Patients with Stable Chronic Obstructive Pulmonary Disease

**DOI:** 10.1155/2016/4173962

**Published:** 2016-04-03

**Authors:** Caimei Tan, Huijuan Huang, Jianquan Zhang, Zhiyi He, Xiaoning Zhong, Jing Bai

**Affiliations:** Department of Respiratory Medicine, First Affiliated Hospital of Guangxi Medical University, Nanning, Guangxi 530021, China

## Abstract

*Objective.* To study the effects of low-dose and long-term treatment with erythromycin on IL-17 and IL-23, in peripheral blood and induced sputum, in patients with stable chronic obstructive pulmonary disease (COPD).* Methods*. Patients were randomly divided into placebo-treated group, group A (12 months of additive treatment with erythromycin, *N* = 18), and group B (6 months of additive treatment with erythromycin followed by 6 months of follow-up, *N* = 18). Inflammatory cells in induced sputum, pulmonary function, and the 6-minute walk distance (6MWD) were analyzed. Concentrations of IL-17 and IL-23 in peripheral blood and sputum were measured using enzyme-linked immunosorbent assays.* Results*. After treatment, sputum and peripheral blood concentrations of IL-17 and IL-23 significantly decreased in groups A and B compared with placebo-treated group. There were no significant differences after erythromycin withdrawal at months 9 and 12 in group B compared with placebo-treated group. An increase in 6MWD was observed after treatment.* Conclusions*. Erythromycin was beneficial and reduced airway inflammation in COPD patients. Underlying mechanisms may involve inhibition of IL-17 and IL-23 mediated airway inflammation. COPD patients treated with erythromycin for 6 months experienced improved exercise capacity. Finally, treatment for 12 months may be more effective than treatment for 6 months.

## 1. Introduction

Chronic obstructive pulmonary disease (COPD) is a chronic lung disease that results in significant morbidity and mortality worldwide [1, 2]. It is characterized by chronic inflammation of the airways and airflow obstruction. The mechanisms underlying COPD progression are complex and involve inflammatory and immune responses. Inflammatory cells, predominantly CD8-positive T cells, neutrophils, and macrophages, are the predominant mediators of inflammation in COPD patients. Neutrophils in particular are considered to play an active role in disease progression. Recently, both animal and clinical studies have demonstrated that IL-17 and IL-23 are closely associated with COPD development and progression [3, 4]. One study demonstrated that the number of IL-23 positive immune cells was higher in the epithelium of severe COPD patients compared to control nonsmokers [3]. Additionally, IL-17A and IL-17F may recruit neutrophils indirectly and play an important role in chronic pulmonary inflammation [5, 6].

With the extensive therapeutic use of corticosteroids in patients with COPD, tolerance has become more common; therefore, alternative anti-inflammatory approaches are needed [7]. Since erythromycin was successfully utilized as a therapy for diffuse panbronchiolitis (DPB) in the late 1980s, the application of long-term erythromycin to the treatment of chronic obstructive pulmonary diseases has received increased attention worldwide and has shown positively curative effects. There have been many studies investigating the application of erythromycin to COPD therapy, indicating effects on prevention of disease exacerbation, antimicrobial activity, and the health status of COPD patients [8, 9]. In the 2015 American College of Chest Physicians and Canadian Thoracic Society Guideline for the prevention of acute exacerbations of COPD, the use of a long-term macrolide to prevent acute exacerbations was suggested for patients with moderate to severe COPD, who have a history of one or more moderate to severe exacerbations in the previous year despite using an optimal maintenance inhaler [10]. However, the mechanisms underlying these effects were unclear. Long-term and low-dose treatment with macrolides was widely used to treat chronic inflammatory lung diseases. The mechanisms underlying the effectiveness included inhibition of neutrophil activation and chemotaxis, which accelerated apoptosis and the elimination of neutrophils, improved phagocytosis by macrophages, and downregulation of inflammatory cytokines (IL-6, IL-8, tumor necrosis factor- (TNF-) *α*, and TNF-*β*) [11, 12]. In a previous study involving a rat model of COPD, our group showed that erythromycin decreased the number of neutrophils and the concentrations of IL-6 and TNF-*α* in bronchoalveolar lavage fluid (BALF) [13]. These results indicated that erythromycin might have anti-inflammatory and immunomodulatory activities in addition to antibacterial functions. However, few studies have investigated the effects of low-dose and long-term treatment with erythromycin on IL-17 and IL-23 mediated airway inflammation in COPD patients. In order to further explore the effects of erythromycin on IL-17 and IL-23 mediated airway inflammation in COPD patients, stable COPD outpatients (GOLD II–IV) were treated with a low oral dose of erythromycin for 6 and 12 months and the effects of treatment on inflammatory cells, IL-17, and IL-23 concentrations in induced sputum and peripheral blood and pulmonary function measured. We expected that this in-depth study could provide new ideas for the prevention and control of COPD.

## 2. Methods

### 2.1. Study Design

After recruitment and screening, a total of 54 eligible patients with stable COPD were randomly divided into an erythromycin group (*N* = 36) and a control group (*N* = 18). The erythromycin group was subdivided into group A (*N* = 18, treated with 125 mg of oral erythromycin 3 times a day for 12 months) and group B (*N* = 18, treated with 125 mg of oral erythromycin 3 times a day for 6 months followed by 6 months of follow-up). The numbers of inflammatory cells in induced sputum, results of spirometry tests, and the inflammatory indices were recorded at baseline and after 3, 6, 9, and 12 months. All the indices were compared between groups and an intragroup comparison was performed. The original treatment was the same for patients in each group and included supplemental oxygen, treatment with theophylline, and treatment with inhaled bronchodilators and corticosteroids. Other macrolides, histamine antagonists, nonsteroidal anti-inflammatory drugs, and oral glucocorticoid treatment were not allowed.

### 2.2. Patients

We recruited 54 stable COPD outpatients (48 men and 6 women, GOLD stages II–IV) at the First Affiliated Hospital of Guangxi Medical University. Patients provided written informed consent to participate in the study, and the study was approved by the Ethics Committee of the First Affiliated Hospital of Guangxi Medical University (number 2015KY-E-036).

The average age was 68.40 ± 7.45 years (range, 49–79 years) and the forced expiratory volume 1 (FEV1) (% predicted) value was 44.46 ± 12.00%. The inclusion criteria were as follows: (1) stable COPD according to the GOLD diagnostic criteria (GOLD stages II–IV) of 2006 (FEV in 1 second [FEV1] < 80% predicted and FEV1/forced vital capacity (FVC) < 70% after bronchial relaxation); (2) no acute exacerbation; (3) no change in therapeutic schedule; and (4) no treatment with any antibiotics or glucocorticoids in the previous 4 weeks. The exclusion criteria included the following: (1) patients with bronchial asthma, primary bronchiectasis, diffuse panbronchiolitis (DPB), active tuberculosis, lung cancer, pneumoconiosis, or other lung diseases with restrictive ventilatory impairment and (2) patients with other serious systemic illnesses such as cardiovascular, nervous, or endocrine system illnesses, blood, hepatic, or kidney diseases, and malignant tumors; (3) patients who were not cooperative or were completely unable to communicate; and (4) patients who experienced serious adverse reactions to erythromycin.

### 2.3. Materials

Enteric coated erythromycin tablets (Lot number: 20060402, 125 mg × 24 tablets/box) were obtained from Dalian Metro Pharmaceutical Company (Dalian, China), dithiothreitol (DTT) was obtained from Shanghai Generay Biotechnology Corporation (Shanghai, China), human enzyme-linked immunosorbent assay (ELISA) kits for IL-17 and IL-23 were obtained from R&D Systems (Minneapolis, MN, USA), the MULTISKAN NK3-mode Microplate Reader was obtained from Thermo Scientific (Waltham, MA, USA), and the Masterscreen*™* PFT system 601-1/IP20 was obtained from BD Biosciences (Yaeger, Germany).

### 2.4. Processing of Peripheral Blood Samples

Peripheral venous blood samples (5 mL) from each patient were collected in heparin-coated anticoagulant tubes. Plasma was separated after centrifugation at 1800 ×g for 20 minutes and stored at −80°C for subsequent analysis.

### 2.5. Processing of Sputum Samples

Sputum was induced with inhalation of 3% hypertonic saline. The procedure was performed according to standard techniques described previously [14]. The induced sputum was collected in sterile test tubes and the volume was measured. An equal amount of 0.1% DTT was added to the collected sputum. The mixture was then placed in a 37°C water bath for 20 minutes and stirred once every 5 minutes for dissolution. An aliquot of the sputum was spread over a cell count board and the total cell count measured. The remaining fraction was centrifuged at 2000 ×g for 10 minutes and the supernatant was collected in Eppendorf tubes and stored at −80°C for subsequent analysis. The cell layer was then mixed and spread over glass slides for Giemsa staining. Cell counts and classification were performed using a high-power microscope. In order to perform counting and classification, at least 200 inflammatory cells and the presence of ≤80% squamous cells were required or the protocol was repeated. Finally, all sputum was examined within 2 hours of collection [15].

### 2.6. Analysis of Inflammatory Cytokines

The concentrations of IL-17 and IL-23 in peripheral blood and induced sputum were assessed using ELISAs. Experiments were performed according to the manufacturer's protocols and the results were analyzed using the Curve Expert 1.3 software for standard curve analysis.

### 2.7. Evaluation of Pulmonary Function and the Six-Minute Walk Distance

Pulmonary function was evaluated using the Masterscreen PET 601-1/IP20 Respiratory Function Instrument (Germany). A six-minute walk distance (6MWD) test was administered in a 50-meter indoor corridor by an expert investigator and was performed in accordance with the standards of the test.

### 2.8. Statistical Analysis

All statistical analyses were performed using SPSS software (version 16.0). The data for continuous variables were expressed as the mean and standard deviation, and the data were summarized as numbers (percentages). A *P* value < .05 was considered statistically significant. Serial examination of sputum inflammatory cells (total cells, neutrophils, macrophages, and lymphocytes) was performed, and the concentrations of inflammatory markers (IL-17 and IL-23) in peripheral blood and sputum over the 12-month period were compared using a four-way repeated-measures variance of general linear model analysis using the number of visits as the repeated measurement and the numbers of inflammatory cells or inflammatory markers as the dependent variable. If Mauchly's test of sphericity was dissatisfied, epsilon correction coefficient was needed or conducted by multivariate variance analysis. Within-subject factor comparisons were performed using repeated-measures ANOVA. Post hoc tests utilizing the Bonferroni correction were conducted whenever the results of the repeated-measures variance analysis indicated statistical significance. The correlations between inflammatory cytokines including IL-17 and IL-23 in both induced sputum and serum and sputum inflammatory cell ratios including neutrophil and macrophage ratios were analyzed using Pearson's Correlation. Baseline parameters were analyzed using one-way analysis of variance or the Friedman tests based on the type of data and homogeneity of variance.

## 3. Results

### 3.1. Clinical Data

There were no significant differences between the 3 groups at baseline with respect to age, gender, smoking history, body mass index, and current medications (i.e., inhaled corticosteroid, theophylline, inhaled anticholinergic agents, or inhaled *β*2-adrenergic agonists) (Table 1).

### 3.2. Sputum Differential Cell Counts

**Figure 1:**
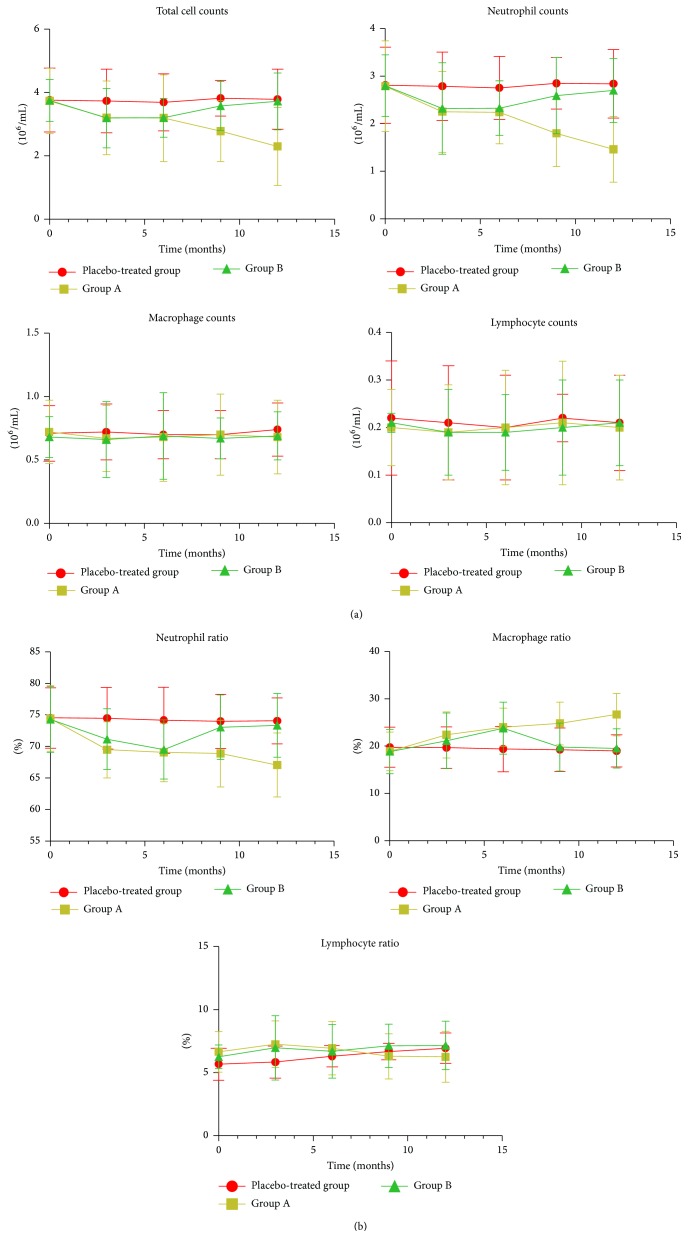
(a) The effects of erythromycin on the number of inflammatory cells in induced sputum (10^6^/mL) in chronic obstructive pulmonary disease patients at baseline and after 3, 6, 9, and 12 months of treatment. There was a significant reduction in total cell counts and neutrophil counts after erythromycin treatment from baseline to 3, 6, 9, and 12 months in group A (all *P* values < .01) and from baseline to 3 and 6 months in group B (all *P* values < .01). There was a significant decrease in total cell counts and neutrophil counts at all time points in group A (all *P* values < .05) and after 3 and 6 months in group B (all *P* values < .05) compared to the placebo-treated group. (b) The effects of erythromycin on the ratio of inflammatory cells in induced sputum (%) in chronic obstructive pulmonary disease patients at baseline and after 3, 6, 9, and 12 months of treatment. Significant reductions in the neutrophil ratio were observed after erythromycin treatment from baseline to 3, 6, 9, and 12 months in group A (all *P* values < .01) and to 3 and 6 months in group B (all *P* values < .01). Compared with placebo-treated group, the significant decreases were also observed in the neutrophil ratio after 3, 6, 9, and 12 months of treatment in group A (all *P* values < .05) and after 6 months in group B (*P* = .029). Increases in the macrophage ratio from baseline to 3, 6, 9, and 12 months in group A (all *P* values < .01) and 6 months in group B (*P* = .004) were observed and compared to placebo; similar increases were significant after 6, 9, and 12 months of treatment with erythromycin in group A (all *P* values < .05) and 6 months in group B (*P* = .040).

The sputum differential cell counts were similar at baseline between the 3 groups (Figures 1(a) and 1(b)). Treatment with erythromycin significantly decreased the total cell counts from baseline to 3, 6, 9, and 12 months (all *P* values < .01) in group A and from baseline to 3 and 6 months (all *P* values < .01) in group B. Decreases in the number of total cells after 3, 6, 9, and 12 months in group A and 3 and 6 months in group B were also significant compared to the placebo-treated control group (all *P* values < .05) (Figure 1(a)). In addition, a similar decrease in the neutrophil counts, neutrophils ratio was observed after treatment with erythromycin from baseline to 3, 6, 9, and 12 months (all *P* values < .01) in group A and to 3 and 6 months in group B (all *P* values < .01). The decreases in both the neutrophil counts and neutrophil ratio after 3, 6, 9, and 12 months, in group A (all *P* values < .05), and after 3 and 6 months in neutrophil counts (all *P* values < .01) and 6 months in the neutrophil ratio (*P* = .029) in group B were significant compared to the placebo-treated group. There was a significant increase in the macrophage ratio from baseline to 3, 6, 9, and 12 months in group A (all *P* values < .01) and baseline to 6 months in group B (*P* = .004). Similarly, there were significant increases in the ratio after 6, 9, and 12 months of treatment with erythromycin in group A (all *P* values < .05) and after 6 months in group B (*P* = .04) when compared to the placebo group (Figure 1(b)). Meanwhile, there were no significant differences shown in total cell counts, neutrophil counts, neutrophil ratio, and macrophage ratio after 9 and 12 months in group B (all *P* values > .05). There were no significant differences in the lymphocyte counts, lymphocyte ratio, and macrophage counts during treatment with erythromycin between the 3 groups (Figures 1(a) and 1(b)).

### 3.3. Inflammatory Cytokines in Sputum

**Figure 2:**
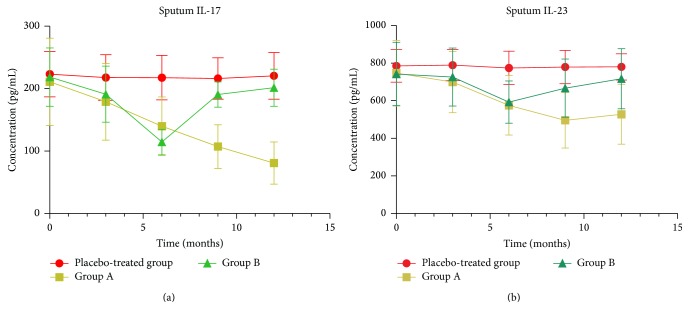
The effects of erythromycin treatment on the concentrations (pg/mL) of IL-17 and IL-23 in sputum from patients with chronic obstructive pulmonary disease at baseline and after 3, 6, 9, and 12 months of treatment. Erythromycin significantly decreased the levels of IL-17 in the sputum after 6, 9, and 12 months of treatment in group A (all *P* values < .001) and after 6 months in group B (*P* < .001) compared to the placebo-treated group. A similar change was observed in IL-23 in patient sputum after 6, 9, and 12 months (all *P* values < .001) in group A and after 6 months of treatment (*P* < .001) in group B compared to the placebo-treated group. Nine and 12 months after erythromycin was discontinued in group B, both IL-17 (*P* = .06 and *P* = .35, resp.) and IL-23 (*P* = .07 and *P* = .63, resp.) in sputum showed no differences compared to the placebo-treated group.

The concentrations of inflammatory cytokines IL-17 and IL-23 at baseline were similar between the 3 groups (Figure 2). Treatment with erythromycin significantly decreased the concentration of IL-17 in sputum from baseline to 6, 9, and 12 months in group A (all *P* values < .01) and from baseline to 3 (*P* = .017) and 6 (*P* < .001) months in group B. When compared to the placebo-treated group, erythromycin significantly decreased the levels of IL-17 in the sputum after 6, 9, and 12 months in group A (all *P* values < .001) and after 6 months in group B (*P* < .001). In addition, there was a significant increase in IL-17 after erythromycin treatment was discontinued after 9 and 12 (all *P* values < .001) months in group B compared to group A for the same study period. However, there were no differences in IL-17 after erythromycin withdrawal at months 9 and 12 in group B compared with the placebo-treated group for the same study period (*P* = .06 and *P* = .35, resp.). Erythromycin treatment also resulted in a significant decrease in the concentration of IL-23 in induced sputum from baseline to 6, 9, and 12 months (all *P* values < .001) in group A and to 6 and 9 months (*P* < .001 and *P* = .010, resp.) in group B. A similar change was observed in IL-23 in patient sputum after 6, 9, and 12 months (all *P* values < .001) in group A and after 6 months of treatment (*P* < .001) in group B when compared to the placebo-treated group. In contrast, there was a significant increase in IL-23, after erythromycin was discontinued at 9 and 12 months (all *P* values < .01) in group B compared to group A for the same study period, and no significant difference compared to the placebo-treated group (*P* = .07 and *P* = .63, resp.) (Figure 2).

### 3.4. Inflammatory Cytokines in Peripheral Blood

**Figure 3:**
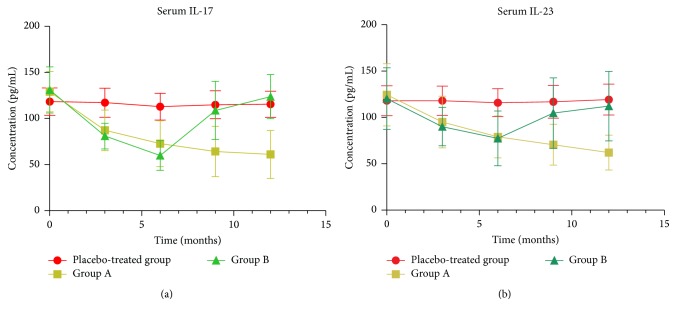
The effects of erythromycin treatment on the concentrations (pg/mL) of IL-17 and IL-23 in chronic obstructive pulmonary disease patients at baseline and after 3, 6, 9, and 12 months of treatment. Erythromycin significantly decreased the levels of IL-17 in serum after 3, 6, 9, and 12 months of treatment in group A (all *P* values < .001) and after 3 and 6 months in group B (all *P* values < .001) compared to the placebo-treated group. A similar change in IL-23 in serum was observed after 3, 6, 9, and 12 months of treatment in group A (all *P* values < .05) and after 3 and 6 months in group B (*P* = .003 and *P* < .001, resp.) compared to the placebo-treated group. After 9 and 12 months (after erythromycin was discontinued) in group B, both IL-17 (*P* > .99 and *P* = .90, resp.) and IL-23 (*P* = .67 and *P* > .99, resp.) in serum showed no differences compared to the placebo-treated group.

The inflammatory indices of serum IL-17 and IL-23 at baseline were similar between the 3 groups (Figure 3). Erythromycin treatment resulted in a significant decrease in the levels of serum IL-17 after 3, 6, 9, and 12 months of treatment (all *P* values < .001) in group A and after 3 and 6 months (all *P* values < .001) in group B compared to the placebo-treated group. A similar change in serum IL-23 was observed after 3, 6, 9, and 12 months of treatment (*P* = .016, *P* < .001, *P* < .001, and *P* < .001, resp.) in group A and after 3 and 6 months of treatment (*P* = .003 and < .001, resp.) in group B compared to the placebo-treated group. There were no significant differences in serum IL-17 (*P* > .99 and *P* = .90, resp.) and serum IL-23 (*P* = .67 and *P* > .99, resp.) after 9 and 12 months (after erythromycin was discontinued) in group B compared to the placebo-treated group.

### 3.5. Pulmonary Function and Six-Minute Walk Distance Tests

**Figure 4:**
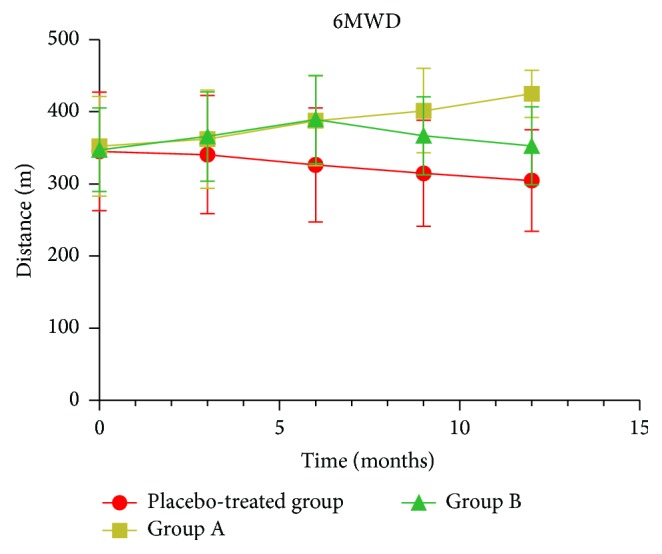
The effect of erythromycin on six-minute walk distance (6MWD, m) in chronic obstructive pulmonary disease patients at baseline and after 3, 6, 9, and 12 months of treatment. Erythromycin did significantly improve the 6MWD after 6, 9, and 12 months of treatment in group A (all *P* values < .05) and after 6 months of treatment in group B (*P* = .038) compared to the placebo-treated group.

All of the pulmonary function indices were similar between the 3 groups at baseline (Table 2). After treatment with erythromycin, pulmonary function indices including the FEV1 (L), FEV1 (% predicted), FVC (L), FEV1/FVC, inspiratory capacity (L), residual volume/total lung capacity, transfer coefficient for carbon monoxide (TLCO, %), and TLCO/alveolar volume did not significantly change during treatment or between the 3 groups (Table 2). Treatment with erythromycin improved the 6MWD from baseline to 3, 6, 9, and 12 months in group A (all *P* values < .01) and to 6 and 9 months in group B (all *P* values < .01) (Table 2, Figure 4). In addition, the increases at 6, 9, and 12 months in group A were also significant compared to the placebo-treated group (*P* = .046, *P* = .001, and *P* < .001, resp.), and a similar increase after 6 months in group B was observed compared to the placebo-treated group (*P* = .038). No significant difference was observed after 9 and 12 months in group B compared to the placebo-treated group. Following placebo treatment, the 6MWD decreased significantly after 6, 9, and 12 months compared to baseline (all *P* values < .01) (Table 2, Figure 4).

### 3.6. Correlation Analysis

**Figure 5:**
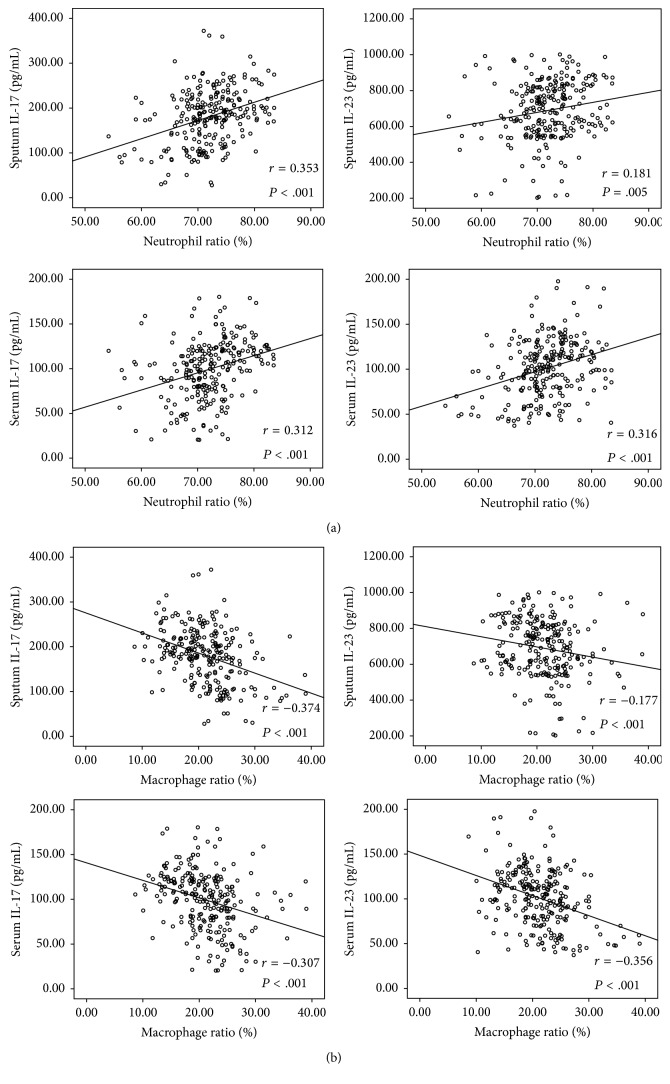
(a) Correlation between inflammatory cytokines including IL-17 and IL-23 in both sputum and serum and sputum neutrophil ratios. (b) Correlation between inflammatory cytokines including IL-17 and IL-23 in both sputum and serum and sputum macrophage ratios.

The correlations between inflammatory cytokines including IL-17 and IL-23 in both induced sputum and serum and different sputum inflammatory cell ratios including neutrophil ratios and macrophage ratios were analyzed using Pearson's Correlation. There were positive correlations between IL-17 in both induced sputum and serum and the sputum neutrophil ratio (*r* = 0.353 and 0.312, resp.; all *P* values < .001) (Figure 5(a)). In addition, similar positive correlations were also observed between IL-23 in both induced sputum and serum and sputum neutrophil ratio (*r* = 0.181 and 0.316, resp.; all *P* values < .001) (Figure 5(a)). However, there were negative correlations between inflammatory cytokines including IL-17 (*r* = −0.374 and −0.307, resp., all *P* values < .001) and IL-23 (*r* = −0.177 and −0.356; *P* = .005 and < .001, resp.) in both induced sputum and serum and the sputum macrophage ratio (Figure 5(b)).

### 3.7. Adverse Events

Five patients withdrew from the study. One patient in group A withdrew because of abdominal pain after treatment with erythromycin. One patient in group B withdrew because of complications (left-sided heart failure). Three patients in the placebo group withdrew from the study (2 had respiratory insufficiency and 1 for an unknown reason).

## 4. Discussion and Conclusion

This randomized controlled study is the first to investigate the effects of low-dose and long-term treatment with erythromycin on the cytokines IL-17 and IL-23 in peripheral blood and induced sputum from patients with COPD. We found that erythromycin had a beneficial effect on pulmonary inflammation in patients with COPD. We observed a significant reduction in the total number of cells, neutrophil counts, and neutrophil ratio in induced sputum from COPD patients after commencement of erythromycin treatment. Previous work has shown a decrease in the neutrophil counts following macrolide treatment in patients with other chronic airway diseases such as DPB [16] and asthma [17]. In a study by Basyigit et al., significant decreases in the total cell count in induced sputum and in the levels of IL-8 and TNF-*α* in patients (all male) with mild to moderate COPD were observed after 14 days of treatment with clarithromycin [12]. The current study demonstrates that macrolides enhance the ability of systemic macrophages to clear apoptotic bronchial epithelial cells [18]. Hodge et al. previously showed that azithromycin was able to improve the ability of alveolar macrophages to phagocytose apoptotic cells. This demonstrates that azithromycin may reverse the reduced ability of alveolar macrophages and monocyte-derived macrophages to clear bacteria and necrotic cellular debris, which can contribute to inflammation and secondary necrosis [19]. Although we did not observe an increase in the number of macrophages in induced sputum after treatment with erythromycin, a significant increase in the sputum macrophage ratio was observed. The results of our study are similar to those of previous studies described above. Erythromycin treatment may reduce the number of neutrophils in induced sputum by promoting phagocytosis of apoptotic cells. Despite their anti-inflammatory activities, macrolides may also play an important role in modulating immune activity.

Blasi et al. found that treatment with azithromycin for 6 months resulted in a decrease in the levels of IL-6 and TNF-*α* in patients with COPD [11]. Similar results were also observed in a study by Basyigit et al. [12]. In our study, decreases in the concentrations of IL-17 and IL-23 in induced sputum and peripheral blood were observed following erythromycin treatment in patients with COPD. We considered that inhibition of IL-17 and IL-23 could be one of the mechanisms by which erythromycin may reduce airway inflammation in patients with COPD. The nature of the immune reaction in COPD indicates that IL-17 and related cytokines may contribute to disorders of immunity [20]. IL-17 has been closely associated with a subject of T helper cells such as Th17 cells, gamma-delta (*γδ*) T cells, natural killer (NK) T cells [21], and neutrophils that have also been shown to produce IL-17A in the lung [22]. In our study, positive correlations were observed between IL-17 and IL-23 in both induced sputum and serum and the neutrophil ratio, while negative correlations were found between IL-17 and IL-23 in both sputum and serum and sputum macrophage ratio. This demonstrates that the decrease in IL-17 and IL-23 levels might be closely related to the reduction in neutrophil accumulation and improvement of phagocytic function of macrophages. However, the concentrations of IL-17 and IL-23 in the induced sputum showed no significant decrease after 3 months of erythromycin treatment in patients with COPD compared to placebo-treated group, while a decrease in the total cell number, neutrophil counts, and concentration of IL-17 in serum was observed after 3 months of treatment. Thus, more meaningful inflammatory indices showed a distinct decrease after 6 months of treatment compared to 3 months.

The results indicate that at least 6 months of maintenance therapy with low-dose erythromycin was required to achieve an appropriate therapeutic effect on airway inflammation. However, additional studies must be performed to confirm these findings. We also found that once erythromycin was discontinued, inflammatory markers increased back to baseline. This finding was similar to the study by Blasi et al. They observed a decrease in the concentrations of TNF-*α* and IL-6 in EBC after 6 months of treatment in COPD patients with azithromycin; however, the concentrations then increased back to baseline once azithromycin treatment was discontinued [11]. We also found that the reduction in inflammatory markers was more significant after 12 months of erythromycin treatment compared to 6 and that the levels of these related indices remained relatively stable. The results indicate that treatment of COPD patients with a low-dose of erythromycin is a long-term process.

COPD is a disease that is characterized by chronic pulmonary inflammation; therefore, anti-inflammation therapy should be administered as long as the inflammation persists. Low-dose erythromycin treatment in patients with stable COPD for 12 months may be more effective than treatment for 6 months. Interestingly, studies that have investigated treatment with macrolides in patients with COPD have produced inconsistent results. In a study by Banerjee et al., no significant effects on the total cell count in sputum, neutrophil count, IL-8, leukotriene B4, TNF-*α* levels, or neutrophil elastase were observed in patients with COPD after 3 months of treatment with clarithromycin [23]. However, clarithromycin did cause a small decrease in the neutrophil differential.

Seemungal et al. also observed no significant differences between the macrolide and placebo arms of the study in measurements including stable FEV1, IL-6 in sputum, IL-8, myeloperoxidase, bacterial flora, or C-reactive protein in serum after treatment with erythromycin for 12 months in COPD patients [8]. Additionally, Fujii et al. and Basyigit et al. showed that treatment with macrolides resulted in a significant decrease in the number of inflammatory cells in sputum and in preinflammatory cytokines in induced sputum, which was consistent with previous studies [12, 16]. Reasons for the inconsistent results regarding the effect of macrolide treatment in chronic inflammatory diseases may be complicated. We hypothesize that differences in the type of macrolide, dosage, and method of administration could explain the differences in the results.

The clinical effects of long-term and low-dose macrolide treatment on patients with DPB have been validated. In 1987, an open trial of low-dose, long-term erythromycin therapy with a 4-year follow-up period was reported. Kudoh et al. showed that treatment with 600 mg of erythromycin for a period of 6 months to 3 years resulted in a significant decrease in the FEV1 [24]. However, the results regarding the effects of macrolides on pulmonary function (i.e., FEV1) were inconsistent. Some studies have reported no improvement in pulmonary function in patients with chronic lung diseases after treatment with macrolides [8, 25]. In our study, we found no significant differences in pulmonary function parameters including FEV1, FVC, IC, and FEV1 (% predicted) between the 3 groups over the 12-month period. We could not confirm whether erythromycin is able to improve pulmonary function in patients with COPD. However, we did observe a significant increase in the 6MWD after treatment with erythromycin and this effect persisted even after treatment was discontinued. This data may indicate that erythromycin treatment administered at a low-dose and long-term may improve the exercise capacity of COPD patients to a certain extent. It is worth noting that though long-term macrolide therapy is generally safe, there are associated adverse effects such as gastrointestinal complaints, hearing decrement, and left heart failure [26], which must be carefully considered despite the low incidence. Cautions should be recommended before initiation of long-term macrolide therapy, and regular monitoring of liver function, electrocardiogram indices, and heart function is needed during the treatment period. The effect of long-term macrolide resistance therapy patterns should also be considered.

Our study had a few limitations. The sample size was small and the 12-month treatment duration may not have been sufficient to assess pulmonary function completely. Clinical studies with larger patient cohorts and longer durations are therefore necessary to determine whether our findings can be replicated.

In conclusion, this study demonstrates that erythromycin treatment in patients with COPD has beneficial effects. This includes an improvement in airway inflammation, which may involve inhibition of the release of inflammatory cytokines IL-17 and IL-23. A decrease in the levels of these cytokines might be closely related to the reduction in neutrophil accumulation and improvement of phagocytic function of macrophages. Additionally, erythromycin treatment may also improve the exercise capacity of patients with COPD. Finally, treatment with erythromycin for 12 months may be more effective than treatment for 6 months. However, the optimum time for treatment with erythromycin in COPD patients is still not clear. Additional studies are required to further investigate the mechanisms of action of erythromycin, optimum dose, appropriate duration of treatment, and side effects in patients with COPD.

## Figures and Tables

**Table 1:** Patients and baseline characteristics.

Parameters	Control	Erythromycin	
		Group A	Group B
Patients, *n*	18	18	18
Males, female, *n*	16, 2	15, 3	17, 1
Age, years	69.3 ± 7.1	68.8 ± 6.0	67.3 ± 6.8
FEV1 (liters)	1.02 ± 0.41	1.04 ± 0.26	1.08 ± 0.26
FEV1 (% predicted)	42.1 ± 18.6	44.8 ± 13.9	46.5 ± 8.9
FEV1/FVC (%)	48.6 ± 8.4	47.4 ± 8.2	47.3 ± 3.8
FVC (liters)	2.5 ± 0.8	2.3 ± 0.6	2.4 ± 0.4
Smoking (pack-years)	41.1 ± 19.8	38.8 ± 28.6	43.9 ± 29.1
Body mass index (kg/m^2^)	23.2 ± 3.1	24.4 ± 3.0	23.1 ± 4.0
Current treatment, *n* (%)			
Inhaled corticosteroid	8 (44.4)	8 (44.4)	7 (38.9)
Theophylline	11 (61.1)	10 (55.6)	10 (55.6)
Inhaled anticholinergic	9 (50.0)	10 (55.6)	9 (50.0)
Inhaled *β*2-adrenergic agonists	13 (72.2)	12 (66.7)	12 (66.7)

The mean ± standard deviation is shown. No significant differences were observed between the 3 groups.

**Table 2:** The effect of erythromycin treatment on pulmonary function and 6-minute walk distance.

Parameter	Time	Placebo group	Group A	Group B
FEV1	Baseline	1.03 ± 0.25	1.05 ± 0.26	1.10 ± 0.26
	Month 6	0.99 ± 0.26	1.14 ± 0.44	1.07 ± 0.27
	Month 12	1.00 ± 0.26	1.11 ± 0.43	1.07 ± 0.28

FEV1 (% predicted)	Baseline	41.15 ± 11.46	45.64 ± 13.88	46.05 ± 8.96
	Month 6	39.66 ± 9.14	48.26 ± 15.81	47.22 ± 9.62
	Month 12	40.03 ± 8.50	48.58 ± 13.24	45.49 ± 9.09

FVC	Baseline	2.06 ± 0.29	2.38 ± 0.59	2.37 ± 0.44
	Month 6	1.97 ± 0.40	2.43 ± 0.70	2.37 ± 0.50
	Month 12	2.04 ± 0.39	2.41 ± 0.76	2.43 ± 0.53

FEV1/FVC	Baseline	49.74 ± 7.96	48.15 ± 7.74	47.38 ± 3.89
	Month 6	50.56 ± 7.50	48.22 ± 8.11	46.63 ± 8.60
	Month 12	49.29 ± 7.56	47.98 ± 8.45	44.91 ± 7.02

6MWD (m)	Baseline	345.09 ± 82.06	352.34 ± 69.05	347.45 ± 58.03
	Month 6	326.37 ± 79.25^#^	387.54 ± 62.35^*∗*#^	389.43 ± 61.26^*∗*#^
	Month 12	304.86 ± 70.55^#^	425.07 ± 32.84^*∗*#^	352.80 ± 53.87

The mean ± standard deviation values are shown. Some pulmonary function indices including the forced expiratory volume in 1 second (FEV1), FEV1 (% predicted), FEV1/forced vital capacity (FVC), FVC, and the 6-minute walk distance (6MWD) are shown. Compared to the placebo treatment, ^*∗*^ *P* value < .05; compared to baseline, ^#^ *P* value < .01.
